# Implementation of a Multi-Agent Carbon Emission Reduction Strategy under the Chinese Dual Governance System: An Evolutionary Game Theoretical Approach

**DOI:** 10.3390/ijerph17228463

**Published:** 2020-11-16

**Authors:** Wenke Wang, Xiaoqiong You, Kebei Liu, Yenchun Jim Wu, Daming You

**Affiliations:** 1 Business School, Sichuan Normal University, Chengdu 610101, China; wangwk@sicnu.edu.cn (W.W.); 2017190251@stu.sicnu.edu.cn (X.Y.); 2018190920@stu.sicnu.edu.cn (K.L.); 2 Sichuan Provincial Key Laboratory of Sci-tech Finance and Mathematical Finance, Sichuan University, Chengdu 610064, China; 3 Graduate Institute of Global Business and Strategy, National Taiwan Normal University, Taipei 10645, Taiwan; 4 College of Management, National Taipei University of Education, Taipei 10645, Taiwan; 5 School of Business, Central South University, Changsha 410083, China

**Keywords:** central-local dual governance, carbon emission reduction, evolutionary game theory, multi-agent, numerical simulation

## Abstract

A central-local dual governance system is the basic system of environmental governance in China. Co-governance between the central environmental protection department (CEPD) and local environmental protection departments (LEPDs) is an important means to effectively promote China’s carbon emission reduction strategy. Accordingly, this paper discusses their interactive decision-making and investigates how to optimize the strategic relationships between the CEPD, LEPDs, and carbon emission enterprises (CEEs) under the dual governance system by constructing a trilateral evolutionary game model and analyzing evolutionary stability strategies, achieving a numerical experiment simulation of evolution processes and determining the impacts of various factors using MATLAB, leading to several countermeasures and suggestions. The results indicate that the CEPD should rationally use the incentive mechanism for LEPDs, improve the carbon tax system, and further penalize the nepotistic relationship of LEPDs and CEEs. Furthermore, it is essential to reform the current LEPD performance evaluation system and reduce the cost of LEPD positive regulation through subsidies and financial transfer payments. Additionally, the CEE strategy is affected by carbon reduction tax rates, penalties, subsides, and emission reduction costs and revenues. This study reveals the consequences of interactions between CEPD, LEPDs, and CEEs and presents options for the redesign of incentive and regulatory mechanisms to improve carbon emission reduction performance in China.

## 1. Introduction

In recent years, due to the frequent occurrence of extreme global weather disasters resulting from greenhouse gas emissions, governments of various countries have continued to vigorously promote and implement carbon emission reduction in various ways [1]. The continuous growth of carbon emissions and increasingly severe environmental pollution have put tremendous pressure on social development in China [2]. The Chinese government has formulated a series of policies and measures intended to reduce carbon emissions, made more stringent carbon emission standards, and promoted the construction of a carbon trading system [3,4,5,6,7]. To achieve the goal of peak carbon emissions around 2030 in order to effectively control total carbon emissions, more stringent environmental protection measures have been implemented, and the National Climate Change Plan (2014–2020) and the Thirteenth Five-Year Plan for Controlling Greenhouse Gas Emissions have been promulgated. The design of a stepped carbon tax system based on carbon emission rights is an attempt to internalize the negative external effects of corporate carbon emissions, gradually increase their production costs, and encourage companies to reduce their carbon emissions or stimulate their adoption of low-carbon green technologies. Most studies show that the carbon emission reduction plan developed by the Chinese government is reasonable and effective [8,9,10,11]. China’s environmental governance and protection currently falls within a dual governance system under the regulation of environmental protection departments at the central and local levels [12]. It is an interactive mechanism and a regulatory model jointly formed by the two levels of government [4], advancing carbon emission reduction and environmental governance [13].

However, the effective implementation of carbon emission reduction policies also requires a series of effective implementation systems, and full consideration of the specific context of policy implementation and the strategic choices of various entities [3]. The existing literature does not clearly acknowledge the incentives and constraints of the dual governance system in terms of carbon emission reduction. The role of China’s central government is precisely to weaken the “yardstick competition” of local provinces by formulating good incentive mechanisms and improving green gross domestic product (GDP) assessment standards [4]. Yardstick competition means that when higher-level governments can use the actions and performance of local governments to evaluate another local government, the local governments will compete in a way that imitates each other [14]. Yardstick competition centers on the “relative performance” comparison to deal with the information asymmetry in the principal–agent framework [15]. The central environmental protection department (CEPD) is the main planner of carbon emission reduction policies and targets. It gives incentives to carbon emission enterprises (CEEs) to reduce the cost and increase their willingness to reduce emissions [3]. The CEPD has provided huge financial support to local environmental protection departments (LEPDs) to increase their decision-making autonomy. The central government holds local governments accountable for environmental protection, reducing the interference of local governments in LEPDs [10].

However, for local governments pursuing economic benefits, in order to ensure the growth of fiscal revenue and investment share in the region, they will be inclined to minimize emission reduction standards [16]. In particular, emission reduction in one region will cause positive externalities in other regions, which makes the emission reduction game between local governments more complicated. In addition, both CEEs and LEPDs have opportunistic tendencies [17]. CEEs do not comply with central carbon emission reduction regulations, and they often even bribe or collude with government officials, while LEPDs regulate such CEE behavior in a negative manner [18]. In this case, the CEPD must review the environmental governance effectiveness of LEPDs and urge them to actively implement regulatory measures. However, the review incurs an economic cost, which will force the CEPD to selectively review the governance effectiveness of the LEPDs, making it difficult for the central environmental protection policy to be observed locally. Besides, carbon emission reduction is poorly motivated due to its positive spillover, hindering the progress of low-carbon economic development. The CEPD and LEPDs, as well as CCEs, dynamically adjust their own strategic options in a carbon emission reduction game system with asymmetric information and conflicting interests [12].

Therefore, the environmental regulation model under the dual governance system will affect the effectiveness of carbon emission regulation to a certain extent [19]. Since the personnel and funds of LEPDs are controlled by local governments, whether they are able to effectively implement the carbon emission reduction policies formulated by the CEPD and actively investigate and punish CEEs for their underlying emissions will not only be subjected to targeting by local governments and review pressure from CEPD, but will also be related to the opportunistic behavior strategies of CEEs sheltered by local governments [3]. In view of this, the strategic interaction between the CEPD, LEPDs, and CEEs needs to be further studied in order to construct a mechanism for effective incentives and regulation in China.

The dual governance literature has made systematic progress in analyzing the incentive mechanism between the levels of Chinese government, but to a large extent it fails to model the strategic dynamics of dual governance behavior [20,21,22]. This research aims to fill this gap using evolutionary game theory and quantitative simulation analysis [23,24]. Evolutionary game theory is used to simulate social dilemmas based on individual cooperation problems, enabling researchers to describe and simulate the results of interactions between game players or groups that collectively affect each other’s benefits [25]. Evolutionary game method is suitable for the study of environmental governance in China; it can quantitatively analyze the iterations and interactions between CEPD, LEPDs, and CEEs and analyze their evolutionary path, providing reform proposals for environmental governance that can be available for reference.

The purpose of this study is to solve three main issues by analyzing the interactions and strategic option results of CEPD, LEPDs, and CEEs. First of all, this study attempts to reveal the mezzanine status of LEPDs as a bridge in the game model, linking the emission reduction decisions of enterprises with CEPD’s environmental regulations. Second, this study aims to understand how the players’ initial strategy options affect the evolutionary path and the convergence speed of the entire system. Third, this study attempts to reveal some specific factors and conditions that affect the tripartite system to reach the ideal state of CEPD inspection, local regulation, and enterprise emission reduction, and the evolutionary path to this ideal state and its impact on the rate of strategic convergence, aiming to provide economic means for the two levels of government to create a low-carbon economy.

The research addresses the above issues. The rest of the paper is structured as follows: Section 2 reviews the relevant literature and identifies the research gap. Section 3 constructs a multi-agent evolutionary game model including assumptions and a pay-off matrix. In Section 4, the replicated dynamic equation is established, and the evolutionary stable strategies of multi-agents are analyzed. Section 5 presents the numerical experimental simulation of evolutionary processes and related parameters and scenario analysis. Final conclusions, suggestions, and limitations of this study are given in Section 6.

This paper mainly makes the following three contributions. First, it provides an understanding of the importance of two levels of government in carbon emission reduction governance under a dual governance system. Evolutionary game analysis reveals how LEPDs’ incentives and penalties affect the efficiency of carbon emission reduction regulation [26,27]. It also stresses that the realization of carbon emission reduction is an ideal state reached by the three parties, rather than just considering the system composed of LEPDs and CEEs.

Second, this research mainly focuses on the strategic interactions and game among multiple agents [12,28]. Advancing multi-agent analysis is helpful in constructing the method logic between evolutionary game theory and the dual governance game, and further enhances the performance of carbon emission reduction by analyzing the multi-agent strategic interaction mechanism under the dual governance system.

Finally, by combining the simulation model with reality, the initial threshold is defined to clarify the sufficient conditions for the three players to converge to the ideal state more quickly. By exploring the factors that influence the evolutionary path, the influencing mechanism among multi-agents is clarified. Consequently, this study explains how to control regulatory costs, rewards and punishments, etc., to promote the realization of the ideal state of central government inspections, local government regulations, and carbon emission reduction for CEEs.

## 2. Literature Review

### 2.1. Dual Governance and Carbon Emission Reduction

Regarding the impact of carbon emission reduction regulatory policies on economic growth, there are mainly two theoretical viewpoints. Neoclassical economics believes that the carbon tax policy is equivalent to adding new restrictions to production decisions, leading to a decline in enterprise performance and market competitiveness [29]. However, the revisionism school, represented by Porter, believes that reasonable environmental regulations can improve the technological innovation capability and productivity of enterprises [30] and help promote long-term economic growth. These two views reflect the two orientations of carbon emission reduction regulatory policies.

It is a clear choice of the central government to make strict carbon emission reduction policies and to force enterprises to transform and upgrade. However, the effects of policy implementation and whether the expected goals can be achieved are also subjected to the policy implementation system and tool selection and the strategic behavior of the implementation subject [31]. In terms of implementation, under China’s dual governance system, the CEPD is responsible for setting carbon emission reduction targets and establishing policy plans, and LEPDs are responsible for implementing policies and inspecting environmental protection performance [4]. The CEPD supervises and evaluates the policy implementation performance of LEPDs [25]. This management system ensures that the central government effectively controls the overall level of carbon emissions while providing local governments with a certain degree of flexibility to balance economic growth with environmental protection.

However, under the dual governance system, the decentralization of economic matters and the centralization of political affairs must be taken into consideration [32]. At present, the dual governance system of economic decentralization and political centralization, the official evaluation system centered on relative economic growth performance, and the dilemma between economic development and environmental regulation are the practical problems faced by local governments in China when implementing environmental regulatory policies [33]. On the one hand, economic competition among local governments has led to insufficient investment in public environmental expenditure and related technology research and development [3]. On the other hand, political promotion championships have made local governments pay more attention to GDP growth, which can lower the environmental protection standards in order to pursue higher economic growth [34]. Additionally, under China’s dual governance system, due to the difference in goals, information asymmetry, and the multidimensional tasks undertaken by local governments, as well as the conflict between the tasks, local governments are motivated to implement the central government’s environmental policies in an incomplete or distorted way in order to maximize their own effectiveness [35]. The lower and incomplete implementation of local governments’ environmental regulatory standards weakens the “innovation compensation” effect of carbon emission policies.

LEPDs, the specific subject of implementing environmental regulation policies, face the “dual governance” of the higher-level CEPD and local governments. They are under the guidance of the central department in business, but subject to the management of local government departments in terms of personnel appointment and fiscal revenue [36]. Under this dual governance system, when local government require LEPDs to relax restrictions on environmental regulation and law enforcement for economic growth performance, LEPDs will have to cooperate in order to get political promotion and departmental interest.

CEEs were generally faced with the pressure of carbon emission reduction driven by local governments [37]. The low-cost, effective supervision of LEPDs can prompt CEEs to comply with environmental regulations [38]. By reducing the carbon quota and rationally increasing carbon trading prices, the government can stimulate CEEs to reduce carbon emission [39]. Subsidy policies from local governments are also a crucial driving force for CEEs to implement strategies for carbon emission reduction [40]. In addition, carbon tax policies, especially bilateral dynamic taxes, are an effective means to control the carbon emissions of CEEs [41,42]. However, carbon emission reduction has increased CEEs’ production costs. Sheltered by local governments, CEEs do not comply with new carbon emission reduction regulations, and LEPDs do not regulate CEEs in a positive manner [43]. At the same time, the CEPD may not review the performance of LEPDs in order to avoid the cost, although it can lead LEPDs directly. Contradictions, conflicts of interest, and asymmetry of information are the root causes of inconsistent actions and speculative behaviors of various participants in carbon emission reduction under the dual governance system [44,45,46].

Scholars have different views on the issue of China’s dual governance system. Simpson pointed out in a pollutant emission reduction study that the environmental policies adopted by a government play a very important role in enterprise emission reduction [47]. Ge et al. believed that the asymmetric strategic interactions between neighboring cities in the form of imitating competition and racing to the top intensifies the lack of environmental regulation efficiency [48]. The government should guide inter-regional cooperation and play a synergistic role to realize the goal of multilevel emission reduction [49]. Zhang et al. showed that the two levels of government are inefficient to encourage local governments to implement environmental regulations [36]. China also needs to make a set of strict ecological and environmental protection policies and administrative measures in order to achieve sustainable economic development [7].

Existing research on the strategic interaction between environmental governance subjects under the dual governance system provides a solid foundation for this research [12,50]. However, scholars have made little progress in revealing the drivers and interactive mechanism of the effectiveness or efficiency of carbon emission regulation itself. Under ideal conditions, through a reasonable incentive mechanism, the CEPD can make LEPDs do their best to reduce carbon emissions to obtain the greatest benefits or maximize social welfare. LEPDs needs to decide whether to actively implement environment regulations or seek greater profits by colluding with CEEs. Therefore, the effective implementation of carbon emissions reduction is a complex and dynamic tripartite game process.

### 2.2. Application of EGT in Carbon Emission Reduction

In the complex economic environment, it is difficult for the subjects involved in carbon emission reduction to remain completely rational, and their strategic choices will be affected by many factors, such as other game players and the external environment [51]. The evolutionary game model can properly reflect this dynamic learning and evolutionary process. Weibull developed the key rules of evolutionary game theory based on the assumption of limited rationality [52]. The crucial concept is that the interaction of individuals interacts with the ever-changing game environment, and the two are interdependent of each other [53]. Through continuous learning, a balanced strategy will be formed among the game players.

Scholars have begun to use evolutionary game models to study carbon emission reduction behavior. Zhu et al. studied the optimal evolutionary path of low-carbon investment strategies and found that an incentive mechanism based on government subsidies is crucial to achieve co-investment, and effective action should be taken to improve the assessment accuracy and supervisory efficiency [54]. Zhang et al. found that it is crucial to implement dynamic carbon trading pricing policies, and government intervention costs and fines play a certain role in promoting carbon emission reduction [55]. Shan et al. analyzed the strategic choices and behavioral characteristics of carbon emission reduction activities among producers, consumers, and decomposers, and further discussed the influences and decisions of the players [56]. Feng et al. believed that when the fines imposed on manufacturers or the losses to the government are considerable, manufacturers will tend to develop innovative technologies, while the government will be less likely to supervise manufacturers [57].

In addition, scholars have begun to use the evolutionary game method to analyze the main interaction mechanism in carbon emission reduction. It has been proven that the evolutionary game can help predict the persistence mode and path of the government to encourage enterprises to implement carbon emission reduction [12]. Under the environmental regulation of the central government, emission reduction strategies, and interactive evolution of local governments, LEPDs and CEEs have jointly promoted the implementation of carbon emission reduction [5]. The low-carbon game between the multiple agents is vital in pushing forward low-carbon strategies among enterprise groups [25]. In particular, the government’s implementation of a bilateral dynamic tax and subsidy mechanism will more effectively encourage enterprises to implement carbon emission reduction strategies [41].

Obviously, the evolutionary game is a quite promising tool that can be used to analyze the strategic interactions between enterprises and local governments under environmental regulations [58]. However, there are still few studies on strategic decisions among participants in the dual governance system and the impact on carbon emission reduction plans. Currently, the majority of scholars are concerned with the influence of government behaviors, strategies, and other factors on the carbon emission behaviors of CEEs, while ignoring the evolution of the government’s own behavior. In particular, current research generally ignores the influence of the initial environment of carbon emissions on the evolution of the entire emission system and the evolution of each participant’s behavioral strategy. Obviously, under the dual governance system, the CEPD, LEPDs, and CEEs not only have common interests, but also face conflicts of interest and misalignment of the goals of the emission reduction plan. Therefore, this research attempts to use evolutionary game theory to construct a behavioral strategy evolution and regulation game model of the participating subjects of carbon emission under the dual system of central and local governments. The model is then used to analyze the behavioral strategy evolution mechanism of the CEPD, LEPDs, and CEEs by depicting their decision-making and learning process, and discuss the mechanism of optimal decision-making by the CEPD and LEPDs based on the behavioral feedback of CEEs to provide guidance for the effective implementation of carbon emission reduction strategies.

## 3. Evolutionary Game Model

### 3.1. Problem Description and Basic Assumptions

Under the dual governance system, the CEPD formulates unified environmental policies, and LEPDs are responsible for implementing these policies and carbon emission regulations. However, LEPDs may pay more attention to short-term economic development than environmental governance, and they have sufficient capability and information to underreport and deprioritize environmental issues [3,4]. The different tasks undertaken by the two levels of government may lead to inconsistencies in their interests. The collusion between CEEs and LEPDs and the CEPD’s regulatory incentives for LEPDs constitutes a complex game relation between the three players.

**Figure 1:**
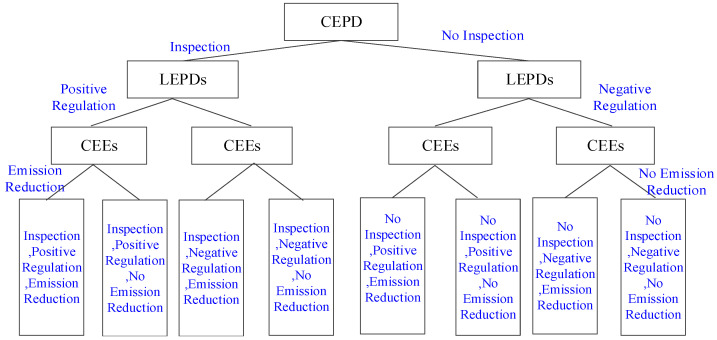
Multi-agent game strategy.

The evolutionary game model can effectively describe the roles of the three players as economic individuals with bounded rationality [12]. These players will generate revenue based on their own initial strategies [4]. Based on checking the respective strategies and revenues of other entities, CEEs, LEPDs, and CEPD repeatedly adjust their behavioral strategies through learning, and finally form more suitable multiple strategies [3,25]. The multi-agent game strategies combined with CEPD, LEPDs, and CEEs are shown in Figure 1 [12,58].

LEPDs have two pure strategic choices: positive regulation or negative regulation. Positive regulation means that in order to protect the environment and maintain public health, LEPDs inspire and restrict the carbon emission reduction activities of enterprises and other market players according to environmental protection laws and regulations and administrative and economic measures [4,12]. Negative regulation refers to the situation where nepotism between CEEs and LEPDs brings greater benefits to LEPDs, or in the case where the gains of positive regulation are less than those of negative regulation, LEPDs tend to choose negative regulation [3]. Then, the initial probabilities of LEPDs choosing positive or negative regulation are *y* and 1 − *y*, respectively, where 0 ≤ *y* ≤ 1.

The CEPD will make the strategic choice of inspection or no inspection based on the previous strategic choices and possible strategic choices of LEPD. “Inspection” means that the CEPD supervises and restricts the regulation performance of LEPDs and urges them to make a strategic choice of positive regulation [5,12]. “No inspection” means that because the cost of collecting and identifying data is higher than the expected gains, the CEPD will forego supervising and inspecting LEPDs [3,4]. At the beginning, the probabilities of adopting the inspection or no inspection strategies are z and 1−z, respectively, where 0≤z≤1.

Finally, taking into account the previous decisions of LEPDs and the CEPD and decisions that may be made in the future, combined with their own interests, CEEs will adopt a strategy of emission reduction or no emission reduction. “Emission reduction” means that CEEs abandon traditional high-carbon production methods and introduce new technologies or adopt innovative low-carbon production activities [4,41]. “No emission reduction” means that CEEs do not implement carbon emission reduction measures in order to achieve greater benefits [3,58]. At the beginning, the probabilities of CEEs choosing emission reduction, or no emission reduction are x and 1−x, respectively, where 0≤x≤1.

### 3.2. Payment Matrix

According to the analysis above, the revenues of CEEs, LEPDs, and CEPD under different behavioral strategy portfolios are as follows:

(1) The revenue of carbon emission reduction obtained by CEEs by implementing low-carbon technological innovation or introducing low-carbon technologies is $M_{1}$, and the corresponding implementation and operating costs are represented as $C_{1}$. When CEEs do not adopt a low-carbon emission reduction strategy, the revenue is $M_{2}$ and the operating costs needed are represented as $C_{2}$. Under positive regulation of LEPDs, the subsidies they provide to CEEs for adopting carbon emission reduction measures is $S_{1}$, and if CEEs do not adopt such measures, the fine imposed by LEPDs is $G_{1}$ [4,12].
(2) When CEEs adopt carbon emission reduction strategies, the potential revenue of LEPDs is $M_{3}$, and the extra regulation costs paid by LEPDs for positive over negative regulation is $C_{3}$. When CEEs do not adopt carbon emission reduction strategies, the governance cost of LEPDs is $C_{4}$. The fine imposed on LEPDs by the CEPD for negative regulation identified during inspection is $G_{2}$ [3,4].
(3) During inspection, the inspection cost paid by CEPD is $C_{5}$ and its gain in reputation is $S_{2}$ [5].
(4) The environmental protection department regulates CEEs’ carbon emission reduction behavior by levying carbon taxes. The carbon tax rate is t, the unit carbon emission of ordinary products is e, the carbon emission rate reduced by increased low-carbon level is ε (0<ε<1), and the unit carbon emission of low-carbon products is e(1−ε). The allocation rate between LEPDs and CEPD regarding the levied carbon tax is *a*. Detailed descriptions of related notations and definitions are shown in Table 1 [3,4,12].

Based on the parameter settings and analysis, eight strategy portfolios of a three-player evolutionary game among CEEs, LEPDs, and CEPD, as well as various matrices under different strategy portfolios, can be constructed, as shown in Table 2 [12,23].

## 4. Analysis of Evolutionary Stable Strategies

### 4.1. Equilibrium Analysis of Evolutionary Game

Evolutionary game theory mainly elaborates the process of continuous optimization of the players’ strategic choices with bounded rationality. It studies the degree of adaptability of any strategy in its set by replicator dynamic analysis, and determines whether the strategy is suitable for evolution in the group and has the stability to resist the invasion of other strategies, focusing on identifying whether the average revenue of the group during the game is less than that of a strategy.

The expected revenues of a CEE choosing to adopt or not adopt carbon emission reduction strategies are $E_{x1}$ and $E_{x2}$, respectively, and the average revenue of a CEE is $E_{x}$.

The expected revenue of a CEE choosing to adopt a carbon emission reduction strategy is [4,23]:

(1) $$E_{x1}=yz\left[M_{1}+S_{1}-C_{1}-et\left(1-\varepsilon \right)\right]+z\left(1-y\right)\left[M_{1}-C_{1}-et\left(1-\varepsilon \right)\right]\;+y\left(1-z\right)\left[M_{1}+S_{1}-C_{1}-\mathrm{et}\left(1-\varepsilon \right)\right]+\left(1-y\right)\left(1-z\right)\left[M_{1}-C_{1}-et\left(1-\varepsilon \right)\right]$$

The expected revenue of a CEE choosing to not adopt a carbon emission reduction strategy is [3,24]:

(2) $$E_{x2}=\mathrm{yz}\left(M_{2}-C_{2}-G_{1}-et\right)+z\left(1-y\right)\left(M_{2}-C_{2}-G_{1}-et\right)\;+y\left(1-z\right)\left(M_{2}-C_{2}-G_{1}-et\right)+\left(1-y\right)\left(1-z\right)\left(M_{2}-C_{2}-et\right)$$

The average revenue of a CEE is [3,4]:

(3) $$E_{x}=xE_{x1}+\left(1-x\right)E_{x2}$$

To achieve the stability condition of the replicator dynamics equation, the constraint conditions need to be satisfied.

The replicator dynamic equation of a CEE is [23,24]:

(4) $$\begin{array}{l} F\left(x\right)=\frac{dx}{dt}=x\left(E_{x1}-E_{x}\right)\\ =x\left(1-x\right)\left[M_{1}+yS_{1}-C_{1}+et\varepsilon -M_{2}+C_{2}+\left(z+y-zy\right)G_{1}\right] \end{array}$$

The expected revenues of an LEPD choosing positive or negative regulation are $E_{y1}$ and $E_{y2}$, respectively, and the average expected revenue is $E_{y}$.

The expected revenue of an LEPD choosing positive regulation is [4,23]:

(5) $$E_{y1}=xz\left[M_{3}-C_{3}-S_{1}+aet\left(1-\varepsilon \right)\right]+x\left(1-z\right)\left[M_{3}-C_{3}-S_{1}+aet\left(1-\varepsilon \right)\right]\;+z\left(1-x\right)\left(-C_{3}-C_{4}+G_{1}+aet\right)+\left(1-z\right)\left(1-x\right)\left(-C_{3}-C_{4}+G_{1}+aet\right)$$

The expected revenue of an LEPD choosing negative regulation is [3,24]:

(6) $$E_{y2}=xz\left[M_{3}+aet\left(1-\varepsilon \right)-G_{2}\right]+x\left(1-z\right)\left[M_{3}+aet\left(1-\varepsilon \right)\right]\;+z\left(1-x\right)\left(-C_{4}+aet-G_{2}\right)+\left(1-z\right)\left(1-x\right)\left(-C_{4}+aet\right)$$

The average benefit of an LEPD is [3,4]:

(7) $$E_{y}=yE_{y1}+\left(1-y\right)E_{y2}$$

The replicator dynamic equation of an LEPD is [23,24]:

(8) $$F\left(y\right)=\frac{dy}{dt}=y\left(E_{y1}-E_{y}\right)=y\left(1-y\right)\left(-C_{3}-xS_{1}+G_{1}-xG_{1}+zG_{2}\right)$$

The expected revenues of CEPD choosing inspection or no inspection are $E_{z1}$ and $E_{z2}$, respectively, and the average revenue of CEPD is $E_{z}$.

The expected revenue of CEPD choosing inspection is [4,23]:

(9) $$E_{z1}=xy\left[-C_{5}+\left(1-a\right)et\left(1-\varepsilon \right)+S_{2}\right]\;+x\left(1-y\right)\left[-C_{5}+\left(1-a\right)et\left(1-\varepsilon \right)+S_{2}+G_{2}\right]\;+y\left(1-x\right)\left[-C_{5}+et\left(1-a\right)+S_{2}\right]\;+\left(1-x\right)\left(1-y\right)\left[-C_{5}+\left(1-a\right)et+S_{2}+G_{2}\right]$$

The expected revenue of CEPD choosing no inspection is [3,24]:

(10) $$E_{z2}=xy\left[\left(1-a\right)et\left(1-\varepsilon \right)\right]+x\left(1-y\right)\left[\left(1-a\right)et\left(1-\varepsilon \right)\right]\;+y\left(1-x\right)\left[\left(1-a\right)et\right]+\left(1-x\right)\left(1-y\right)\left[\left(1-a\right)et\right]$$

The average revenue of CEPD is [3,4]:

(11) $$E_{z}=zE_{z1}+\left(1-z\right)E_{z2}$$

The replicator dynamic equation of CEPD is [23,24]:

(12) $$F\left(z\right)=\frac{dz}{dt}=z\left(1-z\right)\left[-C_{5}+S_{2}+\left(1-y\right)G_{2}\right]$$

According to evolutionary game theory, when the replicator dynamic equation equals zero, the stability can be judged through the equilibrium point.

### 4.2. Stability Analysis of Equilibrium Strategy

#### 4.2.1. Stability Analysis between CEE and LEPD

We use Equations (4) and (8) simultaneously as a replicator dynamic system (I) to study the balance between LEPD and CEE by considering their strategic choices as a replicator dynamic system. Let each equation in the replicator dynamic system (I) be zero, that is, satisfy the conditions F(x)=0,F(y)=0 [23]. On this basis, five equilibrium points of the replicator dynamic equation are obtained: $\left(0,\;0\right),\;\left(0,\;1\right),\;\left(1,\;1\right),\;\left(1,\;0\right),\;\left(x^{*},y^{*}\right)$, with $x^{*}=\frac{G_{1}+zG_{2}-C_{3}}{S_{1}+G_{1}}$ and $y^{*}=\frac{M_{1}-C_{1}+et\varepsilon -M_{2}+C_{2}+zG_{1}}{zG_{1}-S_{1}-G_{1}}$.

Equilibrium points are not all evolutionary stable strategy (ESS), because ESS must also be capable of recovering to a stable point under disturbances. Therefore, according to Friedman (1998), the local asymptotic stability method can be used in the Jacobian matrix to determine the stability of the equilibrium points of an evolutionary system [59]. Using the replicator dynamic equations in Equations (4) and (8) to take the derivatives of x, y and determine the final ESS of the game, we can obtain the Jacobian matrix ($J_{1}$):

$$J_{1}=\left(\begin{array}{c} \frac{\partial X}{\partial x}\;\frac{\partial X}{\partial y}\\ \frac{\partial Y}{\partial x}\;\frac{\partial Y}{\partial y} \end{array}\right)=\left(\begin{array}{c} \pi_{1}\;\pi_{2}\\ \pi_{3}\;\pi_{4} \end{array}\right)$$

$\pi_{1}=\left(1-2x\right)\left[M_{1}+yS_{1}-C_{1}+et\varepsilon -M_{2}+C_{2}+\left(z+y-zy\right)G_{1}\right],$

$\pi_{2}=x\left(1-x\right)\left(S_{1}+G_{1}-zG_{1}\right),\pi_{3}=y\left(1-y\right)\left(-S_{1}-G_{1}\right),$

$\pi_{4}=\left(1-2y\right)\left(-C_{3}-xS_{1}+G_{1}-xG_{1}+zG_{2}\right)$

The determinant (*det*) and trace (*tr*) of $J_{1}$ are:

$$\det J_{1}=\pi_{1}\;\pi_{4}-\;\pi_{2}\pi_{3},\;trJ_{1}=\pi_{1}+\pi_{4}$$

When $\det J_{1}=\pi_{1}\;\pi_{4}-\;\pi_{2}\pi_{3}>0$ and $trJ_{1}=\pi_{1}+\pi_{4}<0$, the ESS state is achieved. At the same time, under the steady state, there must be anti-interference ability, i.e., $\frac{dx}{dt}<0\;,\frac{dy}{dt}>0$. Therefore, according to different parameter ranges, let $w_{1}=M_{1}-C_{1}+et\varepsilon -M_{2}+C_{2}+zG_{1}$, $w_{2}=S_{1}+G_{1}-zG_{1}$, $w_{3}=-S_{1}-G_{1}$ and $w_{4}=-C_{3}+G_{1}+zG_{2}$. Obviously, $w_{2}>0,\;w_{3}<0$. The conclusion is shown in Table 3.

#### 4.2.2. Stability Analysis between LEPD and CEPD

We use Equations (8) and (12) simultaneously as a replicator dynamic system (II) to study the equilibrium between CEPD and LEPD by considering the strategic choices of the two levels of environmental protection governance as a replicator dynamic system [12]. Let F(y)=0,F(z)=0, five equilibrium points are obtained: $\left(0,0\right),\;\left(0,1\right),\;\left(1,1\right),\;\left(1,0\right),\;\left(y^{*},z^{*}\right)$, and with y∈(0,1),z∈(0,1), the result is $y^{*}=\frac{M_{1}-C_{1}+et\varepsilon -M_{2}+C_{2}+zG_{1}}{zG_{1}-S_{1}-G_{1}}$, $z^{*}=\frac{C_{3}+xS_{1}-G_{1}+xG_{1}}{G_{2}}$.

The Jacobian matrix ($J_{2}$) obtained is:

$$J_{2}=\left(\begin{array}{c} \frac{\partial Y}{\partial y}\;\frac{\partial Y}{\partial z}\\ \frac{\partial Z}{\partial y}\;\frac{\partial Z}{\partial z} \end{array}\right)=\left(\begin{array}{c} \pi_{5}\;\pi_{6}\\ \pi_{7}\;\pi_{8} \end{array}\right).$$

Therefore, $\pi_{5}=\left(1-2y\right)\left(-C_{3}-xS_{1}+G_{1}-xG_{1}+zG_{2}\right)$, $\pi_{6}=y\left(1-y\right)\left(-G_{2}\right)$, $\pi_{7}=z\left(1-z\right)\left(-G_{2}\right)$, $\pi_{8}=\left(1-2z\right)\left[-C_{5}+S_{2}+\left(1-y\right)G_{2}\right]$

The determinant (*det*) and trace (*tr*) of $J_{2}$ are:

$$\det J_{2}=\pi_{5}\;\pi_{8}-\;\pi_{6}\pi_{7},\;trJ_{2}=\pi_{5}+\pi_{8}$$

When the conditions of $\det J_{2}>0$ and $trJ_{2}<0$ are satisfied, the locally asymptotically stable fixed point corresponds to the ESS and needs to satisfy $\frac{dy}{dt}<0\;,\frac{dz}{dt}>0$. Similarly, let $w_{6}=-C_{3}-xS_{1}+G_{1}-xG_{1}$ and $w_{7}=-C_{5}+S_{2}+G_{2}$. The conclusion is shown in Table 4 [59].

Based on replicator dynamic systems (I) and (II) in Table 3 and Table 4, the final stability points (ESS) of CEPD, LEPD, and CEE are (0, 0, 0) and (1, 1, 1).

This paper aims to promote the establishment of an ideal governance model combining CEEs’ implementation of carbon emission reduction strategies, LEPDs’ positive regulation, and CEPD’s inspection under a dual governance system. Based on the above analysis, when the condition of $M_{1}+yS_{1}-C_{1}+et\varepsilon -M_{2}+C_{2}+\left(z+y-zy\right)G_{1}>0$ is satisfied, it is possible to make the evolution of x tend toward x=1. By limiting the initial threshold of x and satisfying the evolutionary conditions of $-C_{3}-xS_{1}+G_{1}-xG_{1}+zG_{2}>0$ and $-C_{5}+S_{2}+\left(1-y\right)G_{2}>0$, the three-player game may evolve toward the ideal decision-making state of x=1, y=1, z=1.

## 5. Simulation and Analysis

A numerical analysis with in-depth sensitivity by MATLAB is illustrated to demonstrate how related parameter values affect the evolutionarily stable strategy and convergence trends of CEEs, LEPDs, and CEPD in an empirical setting and to gain managerial insights based on the above theoretical results.

### 5.1. Related Data

The data were mainly derived from China Statistical Yearbook, China Statistical Yearbook on the Environment, and China Energy Statistical Yearbook and references in the field of environmental regulation [3,4]. Due to the diversity of data and the abstractness of variables, some measures were difficult to quantify. The Delphi method was used to quantify the data by relevant experts to make the data dimensionless until the results gradually converged. Finally, an open discussion was conducted, and the data were obtained, as shown in Table 5.

### 5.2. Impact of Initial Strategy on Evolutionary Results

**Figure 2:**
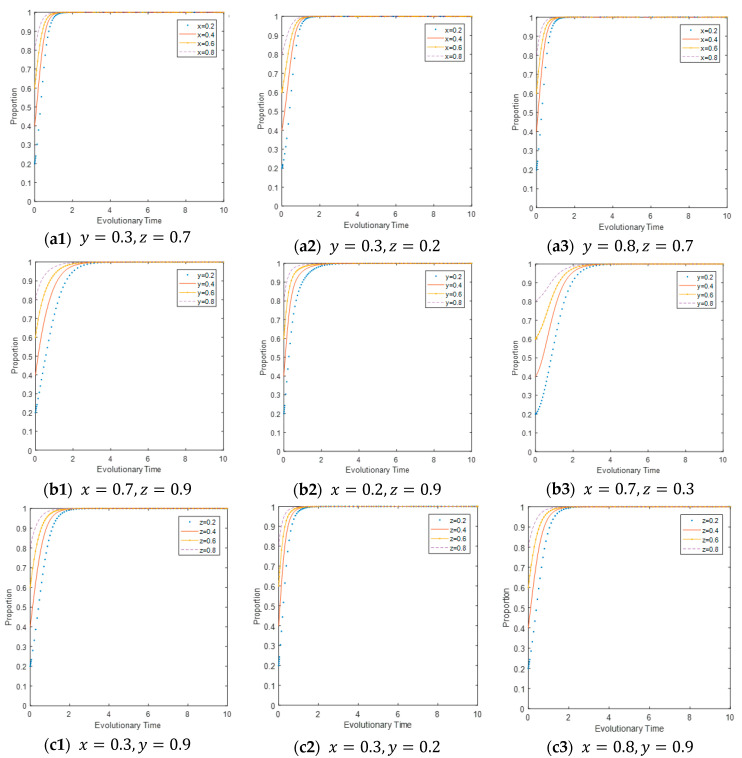
Impact of initial strategy on evolutionary results.

The initial values of y and z were randomly selected. As illustrated in Figure 2(a1–a3), under the condition of fixed y and fixed z, the larger z is, the (slightly) faster x converges to the ideal state, and the greater y is, the sooner x finally converges to the ideal state. This means that the initial strategy of LEPDs and CEPD influences the strategy of CEEs, which will eventually choose to adopt carbon emission reduction strategies.

Simultaneously, when the initial value of x and z are randomly selected, as shown in Figure 2(b1–b3), under the condition of fixed x and fixed z, the larger z is, the faster y converges to the ideal state and the greater x is, the (slightly) faster y approaches 1 but with little change. Therefore, the strategy of LEPDs continues to change with *t* and they will choose to positively regulate.

Moreover, the initial values of x and y were randomly chosen, as illustrated in Figure 2(c1–c3). Under the condition of fixed x and fixed y, the larger y is, the (slightly) faster z approaches 1 but with little change, and the greater x is, the (slightly) faster z approaches 1 but with little change. Then, the strategy of CEPD continues to change and it will choose to inspect. To sum up, as illustrated in Figure 2, the initial probability of each agent affects the evolutionary results of the multi-agents. In addition, each subject is affected by the initial probabilities and strategies of the other two.

### 5.3. Impact of Parameter Change on Evolutionary Results

We analyzed the influence of parameter changes on the evolutionary results with regard to promoting CEEs, LEPDs, and CEPD to form a sustainable regulation mode of carbon emission reduction, aiming to study how effective parameter changes can accelerate the evolution of the system to the ideal state, the point (1, 1, 1).

**Figure 3:**
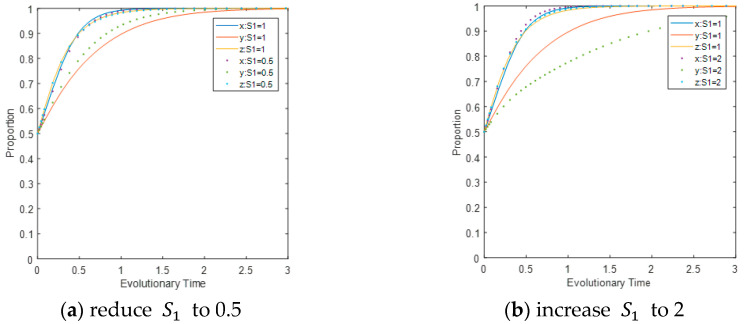
Impact of $S_{1}$ on the evolutionary path of multi-agents.

The influence of subsidies given to CEEs undertaking carbon-reduction activities under LEPD regulation on the evolutionary path. Reduce the value of $S_{1}$ from 1 to 0.5 and increase it to 2 separately, while keeping other parameter settings unchanged. The initial values of *x*, *y*, and *z* are all set as 0.5; i.e., the initial probability of CEEs’ carbon reduction strategy, LEPDs’ regulation, and CEPD’s inspection is 0.5. The evolutionary path of the game system is shown in Figure 3. It can be concluded from the figure that reducing the value of $S_{1}$ alone will not change the direction of the three players’ final convergence to the equilibrium state, and lower subsidies significantly accelerate the convergence of LEPDs to the ideal state, because they increase the expected income of LEPDs. In addition, comparing Figure 3a,b, it can be seen that $S_{1}$ changes with the same amplitude, while LEPDs change with the biggest amplitude. This means that LEPDs are most sensitive to changes. In fact, their higher reliance on subsidies seems to weaken the enthusiasm of the regulatory forces. As LEPDs’ expected revenue declines, the scale of enterprises within their jurisdiction will be further reduced, thereby affecting their profits.

**Figure 4:**
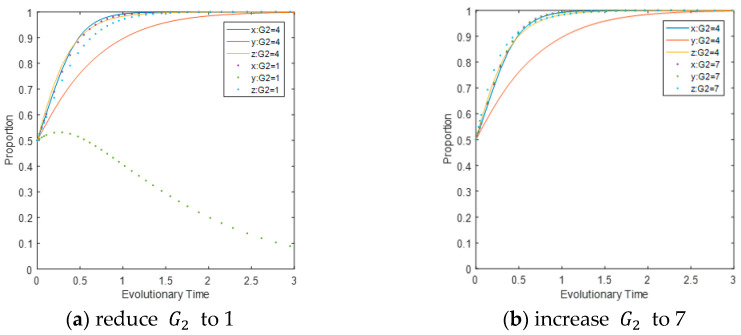
Impact of $G_{2}\;$ on the evolutionary path of multi-agents.

The influence of fines imposed on LEPDs for their deregulation identified during CEPD’s inspection on the evolutionary path. Reduce the value of $G_{2}$ to 1 and increase it to 7 separately, while keeping other parameters unchanged. It can be seen from Figure 4a that reducing the penalty will directly affect the evolutionary direction of the system, leading to a direct change of LEPDs from choosing a regulation strategy to a deregulation strategy. Increasing the penalty shortens the time for the system to become an ESS, as shown in Figure 4b. Comparing Figure 4a,b, it can be seen that LEPDs are the most sensitive to changes. This implies that the reduction in CEPD penalties will reduce the cost of LEPDs’ deregulation, so that they will not regulate CEEs in order to maximize benefits. In addition, the reduction in CEPD penalties will indirectly inhibit the speed of CEEs to carry out carbon reduction activities. Therefore, CEPD can increase the penalties for LEPDs with negative regulation, restrict their behavior through continuous attention and use of laws and policies, and increase their motivation to choose regulatory strategies, thereby encouraging them to actively regulate CEEs’ production activities and achieving the carbon reduction target.

**Figure 5:**
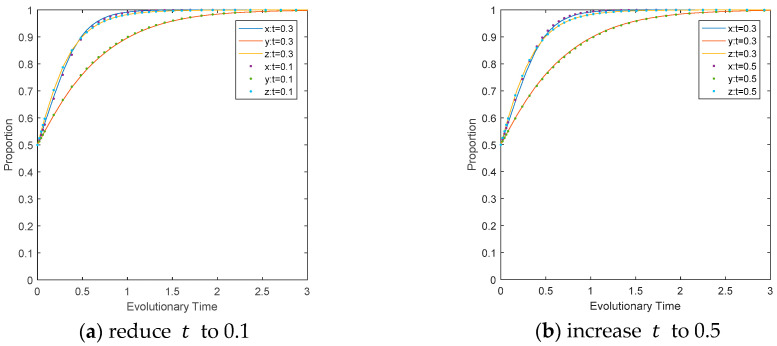
Impact of t  on the evolutionary path of multi-agents.

The influence of the carbon tax rate on the evolutionary path. Carbon tax is an important economic means to control environmental pollution. Converting the external cost caused by CO_2_ emissions into production costs and market prices can redistribute environmental resources and realize CO_2_ reduction. Reduce the value of *t* to 0.1 and increase it to 0.5 separately, while keeping other parameters unchanged. It can be seen from Figure 5 that changes in the carbon tax rate penalty will not affect the evolutionary direction of the system, but an increase of the carbon tax rate will accelerate the evolution of CEEs to the ideal state with less impact on LEPDs and CEPD. This may be because the increased tax rate will impose more economic burden on CEEs, forcing them to choose measures such as introducing new technologies to achieve low-carbon production, thereby reducing their economic burden. The introduction of a carbon tax and an increase in its rate will promote social equity and better guarantee social welfare. Under the high carbon tax environment, CEEs will be more inclined to choose a low-carbon development path, learn to master low-carbon technologies, and improve energy efficiency, thereby promoting the adjustment, optimization, and upgrading of the industrial structure.

**Figure 6:**
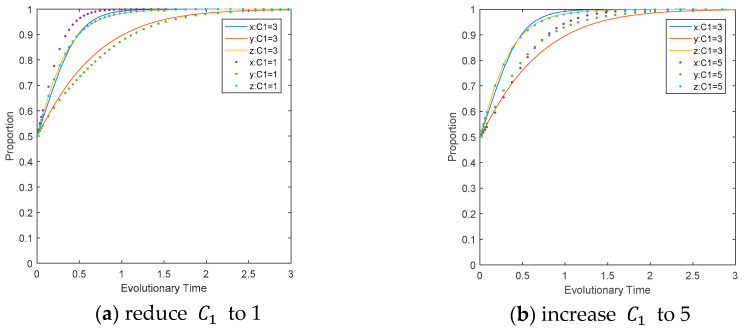
Impact of $C_{1}$ on the evolutionary path of multi-agents.

The influence of the cost paid by CEEs for carbon reduction on the evolutionary path. Change the value of $C_{1}$ while keeping other parameters unchanged, as shown in Figure 6. It can be concluded in Figure 6a that reducing the value of $C_{1}$ will accelerate the speed at which CEE converges to the ideal state, while restraining the speed at which LEPDs converge to ESS. Comparing Figure 6a,b, it can be seen that the response amplitude of the game player to the increase of $C_{1}$ is greater than its decrease, and CEEs show the greatest change amplitude for the same change amplitude of $C_{1}$, i.e., CEEs are the most sensitive to change.

**Figure 7:**
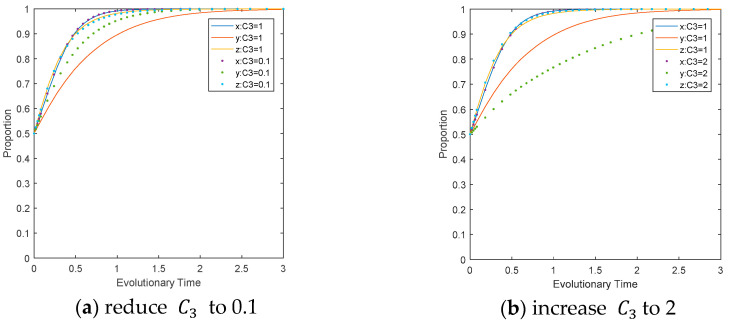
Impact of $C_{3}$ on the evolutionary path of multi-agents.

The influence of LEPD regulation on the evolutionary path. Reduce the value of $C_{3}$ to 0.1 and increase it to 2 separately, while keeping other parameters unchanged, as shown in Figure 7. Comparing Figure 7a,b, it can be seen that the variation in LEPD regulatory cost will not change the evolutionary direction of the entire system, and has little impact on the CEE group and the evolutionary results of CEPD, but an important impact on the speed at which LEPDs converge to ESS. As the regulatory cost increases, LEPDs will slow down the speed of convergence to ESS, while a reduction in regulatory cost will speed up the convergence. This is because the increased regulatory cost and decreased net income of LEPDs undermine the enthusiasm for LEPD regulation.

**Figure 8:**
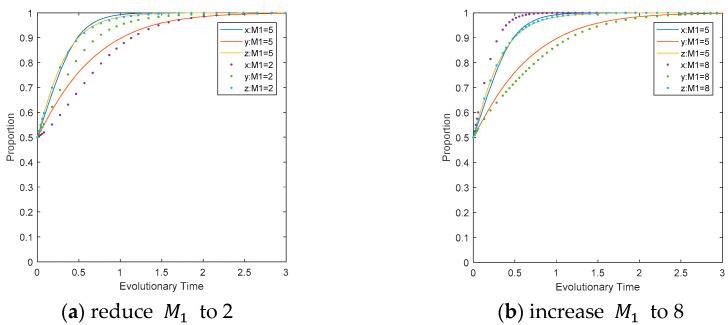
Impact of $M_{1}$ on the evolutionary path of multi-agents.

The influence of revenue from CEEs’ carbon reduction measures on the evolutionary path. Change the parameter setting for the revenue from CEEs’ carbon reduction measures while keeping other parameters unchanged, as shown in Figure 8. Analyze the direct and indirect interference of the changes to the evolutionary path of the three game players. The reduction in revenue from carbon reduction measures will significantly inhibit the speed of CEEs’ convergence to the ideal state and, to a certain extent, can shorten the time for LEPDs to converge to the ideal state. Comparing Figure 8a,b, it can be concluded that with the change of $M_{1}$, CEEs and LEPDs show greater deviations, and CEEs are the most sensitive to changes.

**Figure 9:**
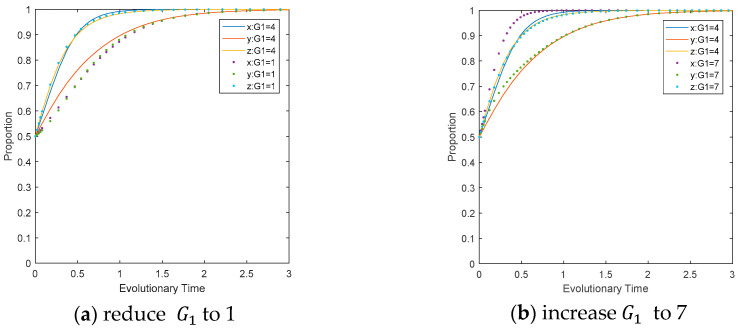
Impact of $G_{1}$ on the evolutionary path of multi-agents.

The influence of penalties imposed on CEEs for failing to carry out carbon reduction activities on the evolutionary path under LEPD regulation. Reduce the value of $G_{1}$ to 1 and increase its value to 7 separately, while keeping other parameter settings unchanged, as shown in Figure 9a,b. Observing the evolutionary results, it can be seen that reducing the value of $G_{1}$ will inhibit the speed at which LEPDs and CEEs converge to the ideal state simultaneously, with little interference to CEPD’s evolution. Comparing Figure 9a,b, it can be seen that the changes in $G_{1}$ will not affect the evolutionary direction of the game system, but the penalties directly interfere with the evolutionary speed of LEPDs and CEEs; especially when the penalty is lower, the changes will affect CEEs and LEPDs more. In addition, CEEs are more sensitive to changes compared to the range of changes of each game player.

### 5.4. Analysis of the Simulation

The results of numerical simulation analysis for the three game players and the complex factors they face have important reference value for the practical application of carbon emission reduction interaction among CEPD, LEPDs, and CEEs. First, the analysis of the dynamic balance of the game reveals that the choice of initial strategy will shape the commitment of multiple players to carbon reduction behaviors. During the evolution process, the interaction of regulatory strategy reflects the continuous adjustment and change based on the interactive games between CEPD and LEPDs.

Second, this study shows that the initial strategic choice probability of the three game players will greatly affect the evolutionary speed. The stronger the willingness of the players to create a low-carbon economy, the more conducive the system will be to the ideal state. The players’ strategy optimization depends on changes of various factors, including reward and punishment mechanism, cost–benefit control, and carbon reduction tax rates. Environmental protection departments can make CEEs choose carbon reduction strategies by changing the influencing factors.

Third, this study reveals the following: Appropriately reducing subsidies for CEEs is more helpful to stimulate the enthusiasm of supervising LEPDs and promote the evolution of the entire system to an ideal state. Appropriately increasing CEPD’s rewards and punishments for LEPDs is a better way to promote the evolution of the system to an ideal state. LEPDs’ penalties on CEEs will probably not change the direction of system evolution. The increase in revenue and total reduction in carbon tax expenditures after CEEs’ low-carbon transformation are higher than the cost of low-carbon transformation and local government fines, and the social benefits earned by CEPD for inspection and the total income from fines are higher than the differences between the inspection costs and carbon tax revenue. The three game players can evolve to a stable state and achieve the ideal low-carbon operation model of CEEs’ active adoption of carbon reduction activities, LEPDs’ positive regulation, and CEPD’s timely inspection.

## 6. Results

### 6.1. Conclusion

Taking the dual governance system as the breakthrough point, the paper builds a three-player evolutionary game model of CEEs, LEPDs, and CEPD, and discusses the process of implementing carbon reduction strategies. By using the evolutionary game theory for solutions, the evolutionary process of the three game players and their ESS are explored. Then, the theoretical results are verified based on numerical simulation to promote the CEEs, LEPDs, and CEPD to a long-term ideal state. Under the dual governance system, the smooth implementation of China’s carbon reduction policies requires the coordination of CEPD and LEPDs. Without minimal central supervision, it seems impossible for LEPDs to insist on increasing carbon reduction or CEEs generating pollution to continue with unrestricted emission strategies over the long term [4,12].

### 6.2. Enlightenment

To promote the effective implementation of China’s environmental regulations and carbon reduction strategies, several policy recommendations seem to be clear and direct.

First of all, from the perspective of policy formulation, CEPD should rationally use the incentive mechanism for LEPDs and improve the carbon tax system to encourage CEEs to implement carbon reduction strategies. Jiang et al. proposed that redesigning the incentive mechanism and optimizing the environmental supervision and governance system will help achieve the ideal decision-making state of environmental governance [12]. We also focus on the interactive decision-making and optimal strategic relationship between CEPD, LEPDs, and CEEs under the dual governance system. Measures such as sewage charges and carbon taxes should be taken for high-carbon industries to speed up their transformation [60]. The amount of carbon taxes and fines shared between CEPD and LEPDs should be rationally divided to inspire the LEPDs to actively enforce the laws. To break down the differentiated carbon reduction tasks, the efficiency of carbon emission and the fairness and historical accumulation of carbon reduction should be considered together to reasonably determine the regional tasks, and more attention should be paid to structural optimization, driving innovation, and accelerating the transformation of society to a low-carbon economy at a reasonable speed.

Second, from the perspective of policy formulation, the reduction in CEPD penalties leads to reducing the cost of LEPDs’ deregulation so that they will not regulate CEEs in order to maximize benefits. In addition, the reduction in CEPD penalties also indirectly inhibits the speed of CEEs to carry out carbon reduction activities. Although the penalties for passive regulation of LEPDs seem to be mature, Chong and Sun concluded that merely increasing political penalties cannot motivate local governments to perform their duties [5]. Previous work confirmed the correctness of our conclusions and research results, and we further proposed to limit the nepotism of LEPDs and CEEs in the form of opportunism by reasonably imposing penalties on LEPDs and encouraging them to implement regulatory policies and enhance the performance of carbon emission reduction. Obviously, the penalties on CEEs imposed by LEPDs directly interfere with the speed at which CEEs carry out carbon reduction activities. Consequently, CEPD can increase the penalties for LEPDs with negative regulation, restrict their behavior through continuous attention and use of laws and policies, and increase their motivation to choose regulatory strategies, thereby encouraging them to actively regulate CEEs’ production activities and achieving the carbon reduction target.

Third, since LEPDs’ strategic choices are affected by political losses and regulatory performance, it is essential to reform the current LEPD performance evaluation system and adopt a more comprehensive system that includes more indicators to evaluate environmental performance. Chen and Chang also mentioned in their work that different measures should be taken according to the fluidity of pollutants, and a novel evaluation system should be constructed [16]. The previous works verified our research findings and we further advised to incorporate environmental regulation, social welfare, etc., into the assessment indicators, and weaken the negative incentives of environmental regulation under the dual governance system.

Fourth, incentives should be provided to local governments that intensely and earnestly implement carbon reduction and environmental regulations, while stricter penalties should be imposed on LEPDs that tolerate corporate pollution [61]. Liu et al. suggested that CEPD’s financial support for and punishment of LEPDs are not the most effective way to achieve the ideal carbon emission reduction development strategy [4]. As mentioned in our research, it is critical to reduce the cost of LEPDs’ positive regulation through subsidies, financial transfer payments, and assistance, ultimately achieving the goals of low carbon activities for CEEs, positive regulation by LEPDs, and CEPD’s low-cost inspection in an optimal manner.

Fifth, the corporate-level CEE strategy seems to have always been affected by carbon reduction tax rates, penalties, and emission reduction costs. Therefore, LEPDs should further increase the penalties for CEEs that do not implement carbon reduction measures while establishing a strong emissions trading system [62]. Wang and Shi suggested that a dynamic punishment mechanism can help governments and enterprises to achieve the ideal stable state [61]. We also conclude that the trading system is expected to promote an effective balance of penalties and further reduce corporate emission reduction costs, thus improving emission reduction efficiency.

Finally, local government departments should actively implement innovation-driven policies to achieve low-carbon transformation and industrial upgrading of enterprises. Zhang et al. illustrated that a reasonable combination of carbon tax and subsidy can influence the choice of green innovation mode [60]. We also mentioned in our research that LEPDs’ subsidies and rewards for CEEs should be focused on technology research and development to maximize the CEEs’ enthusiasm for low-emission innovation, enhancing the revenue of corporate emissions reduction measures, and encourage companies to actively reduce emissions.

### 6.3. Limitations

This research has several limitations. It only explores sufficient stability conditions among the three players, CEPD, LEPDs, and CEEs, in the ideal state of the game, and a summary of the stable equilibrium conditions of the players in more complex situations needs to be further combined with the real research. In addition, in the long run, the implementation of carbon reduction strategies will be a dynamically changing process over time, and the strategic interaction among CEPD, LEPDs, and CEEs will present a more complex dynamic process. The study needs to keep adjusting and optimizing based on more complicated internal and external factors. Finally, the model in our research makes broad and valid recommendations; the research needs to construct the model focused on target situations that share similar socioeconomic backgrounds. These issues create great challenges for future study.

## Figures and Tables

**Table 1:** Descriptions of notations. CEE, carbon emission enterprise; LEPD, local environmental protection department; CEPD, central environmental protection department.

Notation	Description
$M_{1}$	Revenues of a CEE adopting a carbon emission reduction strategy
$C_{1}$	Costs of a CEE adopting a carbon emission reduction strategy
$M_{2}$	Revenues of a CEE that does not adopt a carbon emission reduction strategy
$C_{2}$	Costs of a CEE that does not adopt a carbon emission reduction strategy
$M_{3}$	Potential revenues of an LEPD with a CEE adopting a carbon emission reduction strategy
$C_{3}$	Extra regulation costs paid by LEPD for positive over negative regulation
$C_{4}$	Environmental pollution control costs paid by LEPD due to a CEE’s failure to reduce carbon emissions and high energy consumption
$C_{5}$	Inspection cost of CEPD
$S_{1}$	Subsidy for CEE carbon emission reduction activities under LEPD regulation
$S_{2}$	Reputation obtained during CEPD inspection
$G_{1}$	Fine imposed on a CEE for failure to reduce carbon emission in production under LEPD regulation
$G_{2}$	Fine imposed on LEPD for deregulation identified during inspection by CEPD
t	Carbon tax rate
ε	Carbon emission rate reduced by increased low-carbon level
e	Unit carbon emissions of ordinary products
a	Ratio of carbon tax levied from CEE shared by LEPD

CEEs—carbon emission enterprises); LEPD—slocal environmental protection departments; CEPD—central environmental protection department.

**Table 2:** Behavioral strategy portfolios of CEE, LEPD, and CEPD and their revenue matrix.

	Emission Reduction of CEE (x)		No Emission Reduction of CEE (1−x)	
	Positive regulation of LEPD (y)	Negative regulation of LEPD (1−y)	Positive regulation of LEPD (y)	Negative regulation of LEPD (1−y)
Inspection of CEPD (z)	$M_{1}+S_{1}-C_{1}-e\left(1-\varepsilon \right)t$ $M_{3}-C_{3}-S_{1}+ae\left(1-\varepsilon \right)t$ $-C_{5}+\left(1-a\right)e\left(1-\varepsilon \right)t+S_{2}$	$M_{1}-C_{1}-e\left(1-\varepsilon \right)t$ $M_{3}+ae\left(1-\varepsilon \right)t-G_{2}$ $-C_{5}+\left(1-a\right)e\left(1-\varepsilon \right)t+S_{2}+G_{2}$	$M_{2}-C_{2}-G_{1}-et$ $-C_{3}-C_{4}+G_{1}+aet$ $-C_{5}+\left(1-a\right)et+S_{2}$	$M_{2}-C_{2}-G_{1}-et$ $-C_{4}+aet-G_{2}$ $-C_{5}+\left(1-a\right)et+S_{2}+G_{2}$
No inspection of CEPD (1−z)	$M_{1}+S_{1}-c_{1}e\left(1-\varepsilon \right)t$ $M_{3}-C_{3}-S_{1}+ae\left(1-\varepsilon \right)t$ (1−a)e(1−ε)t	$M_{1}-C_{1}-e\left(1-\varepsilon \right)t$ $M_{3}+ae\left(1-\varepsilon \right)$ (1−a)e(1−ε)t	$M_{2}-C_{2}-G_{1}-et$ $-C_{3}-C_{4}+G_{1}+aet$ (1−a)et	$M_{2}-C_{2}-et$ $-C_{4}+aet$ (1−a)et

CEEs—carbon emission enterprises; LEPDs—local environmental protection departments; CEPD—central environmental protection department.

**Table 3:** Stability analysis between CEE and LEPD.

Equilibrium Point (x,y)	$\det J_{1}$	$trJ_{1}$	Result	Equilibrium Condition
(0,0)	+	−	ESS	$w_{1}<0,w_{4}<0$
(0,1)	+	−	ESS	$w_{1}<0,w_{4}>0$
(1,1)	+	−	ESS	$w_{1}>0,w_{4}>0$
(1,0)	+	+	Unstable	No conditions are stable
$\left(x^{*},y^{*}\right)$	0	0	Saddle point	0, 0

CEE—carbon emission enterprise; LEPD—local environmental protection department.

**Table 4:** Stability between LEPD and CEPD.

Equilibrium Point (x,y)	$\det J_{2}$	$trJ_{2}$	Result	Equilibrium Condition
(0,0)	+	-	ESS	$w_{6}<0,w_{7}<0$
(0,1)	+	-	ESS	$w_{6}<G_{2},w_{7}>0$
(1,1)	+	-	ESS	$w_{6}>G_{2},w_{7}>G_{2}$
(1,0)	+	+	Unstable	No conditions are stable
$\left(y^{*},z^{*}\right)$	0	0	Saddle point	0, 0

LEPD—local environmental protection department; CEP—central environmental protection department.

**Table 5:** Initial simulation parameters.

Variable	$M_{1}$	$M_{2}$	$M_{3}$	$C_{1}$	$C_{2}$	$C_{3}$	$C_{4}$	$C_{5}$	$G_{2}$	$S_{1}$	$S_{2}$	e	t	ε	a	$G_{1}$
**Value**	5	3	2	3	1	1	4	1	4	1	4	2	0.3	0.5	0.3	4
